# The effects of beta-hydoxy-beta-methylbutyrate free acid supplementation on muscle damage, hormonal status, and performance following a high volume 2-week overreaching cycle

**DOI:** 10.1186/1550-2783-9-S1-P4

**Published:** 2012-11-19

**Authors:** GS Davis, Ryan P Lowery, NM Duncan, EM Sikorski, JA Rathmacher, SM Baier, TJ Morrison, KA Dunsmore, MA Naimo, J Walters, J Joy, Stephanie MC Wilson, Jacob M Wilson

**Affiliations:** 1 Department of Health Sciences and Human Performance, The University of Tampa, Tampa, FL, USA; 2 Department of Biology, The University of Tampa, Tampa, FL, USA; 3 Metabolic Technologies, Inc., Iowa State University Research Park, Ames, IA, USA; 4 Department of Animal Science, Iowa State University, Ames, IA, USA; 5 Department of Nutrition, IMG Performance Institute, IMG Academies, Bradenton, FL, USA

## Background

Athletes exposed to extreme training loads such as those that occur during multiple-game tournaments, two a day practices, or sudden increases in volume are prone to overreaching. Beta-hydoxy-beta-methylbutyrate (HMB) is thought to increase regenerative capacity following high intensity exercise. However, to date, its effects on muscle damage, hormonal status, and performance during overreaching have yet to be investigated. Therefore the purpose of this investigation was to determine the effects of HMB free acid (HMB-FA) supplementation on indices of muscle damage, strength, power, and cortisol following a 2-week overreaching cycle.

## Methods

Twenty resistance trained males aged 21.3 ± 1.9 years were recruited for the study and randomly assigned to consume 3 g per day of HMB-FA (combined with food-grade orange flavors and sweeteners) or a placebo (food-grade orange flavors and sweeteners). All subjects were placed on a diet consisting of 25 % protein, 50 % carbohydrates, and 25 % fat by a registered dietician who specialized in sport (RD, LDN, CISSN). Seventy-two hours prior to the overreaching phase subjects were tested for baseline measures of creatine kinase (CK), cortisol, Wingate power and strength on the squat, bench press, and deadlift. Following, all subjects participated in a 2-week high volume resistance-training cycle. Each Monday through Thursday, subjects performed 3 maximal sets of 8-12 repetitions and 60-90 seconds rest of squats, leg press, bench press, deadlifts, pull-ups, military press, bent over rows, barbell curls and extensions. On Friday subjects were given three 1-RM attempts on the squat, bench press, and deadlift for a total of 9 maximal working sets, followed by power testing on the Wingate on Saturday. A 2 X 3 (Group X time (weeks 0, 1, and 2)) repeated measures ANOVA was used to assess any main effects. If main effects were found LSD post hoc tests were incorporated to determine where differences were located.

## Results

Significant group X time effects were found for CK, which relative to baseline values (256.1 ± 28.3 U/L) increased at weeks 1 (493.8 ± 36.3 U/L) and 2 (533.4 ± 49.0 U/L) in the placebo group, but not the HMB group (p<0.05). There were also group X time effects for strength of the squat, bench press, and deadlift, which decreased during weeks 1 and 2 (ranging from -5.6 to -7.1% across strength measures) in the placebo group, but not the HMB group (p<0.05). A group x time effect was found for Wingate peak power, which relative to baseline values (991.0 ± 60.1 watts) was lower at weeks 1 (924.6 ± 58.3 watts) and 2 (946.6 ± 59.1 watts) in the placebo group but not the HMB group. Finally there were group X time effects for cortisol, which relative to baseline (19.3 ± 1.4 ug/dl) increased in both weeks 1 (22.1 ± 1.4 ug/dl) and 2 (23.7 ± 1.0 ug/dl) in the placebo group, but not the HMB group (p<0.05).

## Conclusions

These results suggest HMB-FA given over a 2-week high volume, low recovery training cycle prevents overreaching, as well as the characteristic rise in serum stress hormones and serum indices of muscle damage.

